# Silencing of Homeodomain-Interacting Protein Kinase 2 (HIPK2) Induces Anti-Adipogenic Effects in 3T3-L1 Adipocytes

**DOI:** 10.3390/cimb48080812

**Published:** 2026-08-12

**Authors:** Anil Kumar Yadav, Nivethasri Lakshmana Perumal, Gi-Young Park, Byeong-Churl Jang

**Affiliations:** 1 Department of Molecular Medicine, College of Medicine, Keimyung University, 1095 Dalgubeoldaero, Dalseogu, Daegu 42601, Republic of Korea; yadav127@umn.edu (A.K.Y.); nivi1998@naver.com (N.L.P.); 2 Department of Rehabilitation Medicine, Daegu Catholic University School of Medicine, Duryugongwon-ro 17-gil, Nam-gu, Daegu 42472, Republic of Korea

**Keywords:** HIPK2, 3T3-L1, STAT-3, C/EBP-α, perilipin A

## Abstract

Homeodomain-interacting protein kinase 2 (HIPK2) is an important regulator of various transcription factors and cofactors, which are involved in cell growth, cell death, and embryonic development. Previously, it has been reported that HIPK2 is upregulated during white fat development. However, the expression and functional role of HIPK2 during adipogenesis remain unclear. Here, we investigated the expression and biological function of HIPK2 during the adipogenesis of 3T3-L1 cells. Importantly, protein and mRNA expression of HIPK2 were significantly upregulated in a time-dependent manner during 3T3-L1 preadipocyte differentiation. Notably, distinct pharmacological inhibitors revealed the pivotal roles of p38 MAPK and PKC in the induction of HIPK2 expression during differentiation of 3T3-L1 cells. Moreover, knockdown of HIPK2 significantly reduced lipid storage and triglyceride (TG) levels without cytotoxicity during 3T3-L1 preadipocyte differentiation. At the mechanistic level, HIPK2 knockdown reduced the expression of CCAAT/enhancer-binding protein-α (C/EBP-α), peroxisome proliferator-activated receptor-γ (PPAR-γ), fatty acid synthase (FAS), perilipin A, leptin, and resistin, as well as the phosphorylation of signal transducer and activator of transcription-3 (STAT-3) during 3T3-L1 preadipocyte differentiation. Taken together, these results revealed that HIPK2 expression is significantly upregulated in p38 MAPK- and PKC-dependent manners, and this upregulation of HIPK2 plays a critical role in lipid accumulation during differentiation of 3T3-L1 cells, which is mediated by control of the expression and phosphorylation levels of C/EBP-α, PPAR-γ, STAT-3, FAS, and perilipin A.

## 1. Introduction

Obesity is a metabolic syndrome that is caused by excessive storage of body fat [1,2]. Obesity increases the chance of noncommunicable diseases and could result in comorbid problems and elevated health risks during the COVID-19 pandemic [3]. Overweight and obesity have become a global health concern, with 13% of the adult population reported as being clinically obese [3,4]. Projections indicate that by 2050, 3.33 million children and early adolescents (age 5–14 years) and 3.41 million adolescents (age 15–24 years) will have overweight or obesity [5].

Adipocyte differentiation (adipogenesis) is a developmental process in which preadipocytes with fibroblast-like morphology become adipocyte-like cells [6,7,8]. Adipogenesis is regulated by transcription factors, including CCAAT/enhancer-binding proteins (C/EBPs), peroxisome proliferator-activated receptors (PPARs), and STAT family proteins [9,10,11,12]. Lipogenesis and lipid droplet (LD) maturation require fatty acid synthase (FAS) and perilipin A [13,14]. White adipose tissue (WAT) was once considered only an energy storage organ [15]. However, in addition to serving as a lipid reservoir, WAT is an endocrine organ that produces adipokines, including adiponectin, leptin, and resistin, which modulate adipogenesis, metabolism, insulin secretion and insulin sensitivity, energy metabolism, and homeostasis [16,17]. Dysregulation of adipocyte differentiation and dysfunction plays a pivotal role in the pathogenesis of obesity and associated disorders, such as diabetes and metabolic syndrome [18]. A better understanding of adipogenesis mechanisms will elucidate the pathophysiology of obesity and related disorders.

Recently, we performed RNA sequencing of 3T3-L1 white preadipocytes (D0), differentiating adipocytes (D2), and differentiated adipocytes (D7), and observed that homeodomain-interacting protein kinase-2 (HIPK2) expression was elevated during differentiation. HIPK2 is a member of the HIPK family (HIPK1, HIPK2, HIPK3, and HIPK4) with serine/threonine kinase activity [19]. HIPK1-HIPK3 were initially identified by their ability to bind homeobox factors, while HIPK4 was identified in the human genome based on high homology with the other three members [19,20]. HIPK2 is well characterized. It regulates several transcriptional coactivators, corepressors, and kinases that influence gene expression involved in cell survival, proliferation, DNA damage response, apoptosis, and differentiation [19,21,22,23,24]. Despite these well-characterized functions, the regulation of HIPK2 expression during white preadipocyte differentiation remains elusive. Therefore, we examined the expression and function of HIPK2 during the differentiation of 3T3-L1 preadipocytes using HIPK2 knockdown. Previous studies have demonstrated that several signaling pathways, including p38 MAPK, JNK, PI3K/AKT, and PKC, play important roles in adipocyte differentiation and adipogenesis through regulation of adipogenic transcription factors, lipid accumulation, and adipocyte maturation [9,10,11,12]. In addition, these signaling pathways have been implicated in the regulation of transcriptional activity, mRNA stability, protein stability, and post-translational modifications of multiple cellular proteins, including HIPK2-associated regulatory mechanisms [25,26]. Based on these observations, we also investigated whether inhibition of these pathways could influence HIPK2 expression during adipogenesis in 3T3-L1 cells.

Experimental results from this study revealed that HIPK2 levels are significantly elevated and regulated by p38 MAPK and PKC-kinase. Upregulation of HIPK2 appears to play a critical role in lipid accumulation and regulates key adipogenesis transcription factors (C/EBP-α, PPAR-γ, STAT-3, FAS) and perilipin A during the differentiation of 3T3-L1 cells. Parts of this study were previously included in the first author’s doctoral thesis, which has been substantially revised and expanded with additional analyses and updated experimental data for the present manuscript [27].

## 2. Materials and Methods

### 2.1. Drugs and Antibodies

3-isobutyl-1-methylxanthine (IBMX), dexamethasone, all-rac-α-Tocopheryl acetate (Cat. No.5009520100), and insulin were purchased from Sigma (St. Louis, MO, USA). Control shRNA plasmids (Cat. No. sc-108060) and HIPK2 shRNA plasmids (Cat. No. sc-39050-SH) were purchased from Santa Cruz Biotechnology (Delaware, CA, USA). PD98059 and SB203580 (Cat. No. BML-E1294) were purchased from Enzo (Farmingdale, NY, USA). SP600125 (Cat. No. 420119) was purchased from Calbiochem (Madison, WI, USA). LY294002 (Cat. No. ST- 420) and GF109203X (Cat. No. EI-246) were purchased from Biomol (Hamburg, Germany). Other chemicals used in this study were of analytical grade.

### 2.2. Cell Culture and Differentiation

Murine 3T3-L1 preadipocytes (ATCC, CL-173™, Manassas, VA, USA) were cultured in Dulbecco’s Modified Eagle’s Medium (DMEM; Welgene, Daegu, Republic of Korea) supplemented with 10% heat-inactivated fetal calf serum (FCS; Gibco, Grand Island, NY, USA) and 1% penicillin–streptomycin (Welgene, Daegu, Republic of Korea). Cells were maintained at 37 °C in a humidified incubator with 5% CO_2_. Adipocyte differentiation was performed as previously described [28]. Briefly, 3T3-L1 preadipocytes were grown to confluence and maintained for an additional 2 days after reaching confluence (Day 0, D0). Differentiation was induced by replacing the culture medium with DMEM containing 10% fetal bovine serum (FBS; Welgene, Daegu, Republic of Korea) supplemented with a differentiation cocktail consisting of 0.5 mM 3-isobutyl-1-methylxanthine (IBMX), 0.5 μM dexamethasone, and 5 μg/mL insulin (all from Sigma-Aldrich, St. Louis, MO, USA) for 2 days (D0–D2). On Day 2 (D2), the medium was replaced with DMEM containing 10% FBS and 5 μg/mL insulin for an additional 3 days (D2–D5). From Day 5 (D5) to Day 8 (D8), cells were maintained in DMEM containing 10% FBS, with the medium replaced every other day.

### 2.3. HIPK2 Short-Hairpin RNA (shRNA) Transfection

For HIPK2 knockdown, 3T3-L1 preadipocytes were seeded into 6-well plates and allowed to attach overnight. Cells were transfected with either control shRNA or HIPK2 shRNA using Lipofectamine™ 2000 (Invitrogen, Carlsbad, CA, USA) according to the manufacturer’s instructions. After 6 h of transfection, the medium was replaced with fresh DMEM supplemented with 10% fetal calf serum (FCS), and cells were cultured for an additional 48 h. Subsequently, adipocyte differentiation of control and HIPK2-shRNA-transfected cells was induced using the standard differentiation protocol described above.

### 2.4. Oil Red O Staining

Lipid accumulation was assessed by Oil Red O staining as previously described [29]. Briefly, on Day 8 (D8), differentiated 3T3-L1 cells were fixed with 10% formaldehyde for 2 h at room temperature (RT), rinsed with 60% isopropanol, and air-dried. Cells were then stained with freshly prepared and filtered Oil Red O working solution (Sigma-Aldrich, St. Louis, MO, USA) for 1 h at RT. After staining, the cells were washed three times with deionized water to remove excess dye. Intracellular lipid droplets were visualized using an inverted microscope (Nikon, Tokyo, Japan).

### 2.5. Cell Survival Assay

For cell counting, control and HIPK2-shRNA-transfected 3T3-L1 preadipocytes were seeded into 24-well plates and differentiated as described above. On Day 8 (D8), cells were harvested and stained with trypan blue, and viable (trypan blue-negative) cells were counted using a hemocytometer under a light microscope. Cell counts were performed in triplicate, and data are presented as the mean ± standard error (SE) from three independent experiments.

### 2.6. Measurement of Intracellular TG

Intracellular triglyceride (TG) content was quantified on Day 8 (D8) of adipocyte differentiation using the AdipoRed™ Assay Reagent (Lonza, Basel, Switzerland) according to the manufacturer’s instructions. Briefly, control and HIPK2-shRNA-transfected 3T3-L1 cells were incubated with AdipoRed reagent, and fluorescence was measured at an excitation wavelength of 485 nm and an emission wavelength of 572 nm using a Victor^3^™ multilabel plate reader (PerkinElmer, Waltham, MA, USA).

### 2.7. Western Blotting

At the indicated time points, cells were lysed in RIPA buffer (Sigma-Aldrich, St. Louis, MO, USA) supplemented with a 1× protease inhibitor cocktail. Supernatant fractions were collected. Proteins were separated by SDS-polyacrylamide gel electrophoresis (SDS-PAGE) and transferred to a polyvinylidene difluoride membrane (PVDF, Millipore, Bedford, MA, USA), and incubated with following primary antibodies: C/EBP-α (1:2000; Santa Cruz Biotechnology, sc-61), PPAR-γ (1:2000; Santa Cruz Biotechnology, sc-7272), Perilipin A (1:2000; BioVision, Milpitas, CA, USA, #3948-200), fatty acid synthase (FAS; 1:2000; BD Biosciences, Milpitas, CA, USA, #9452), HIPK2 (1:1000; Santa Cruz Biotechnology, sc-100383), phospho-STAT3 (Tyr705; 1:2000; Santa Cruz Biotechnology, sc-8059), total STAT3 (1:2000; Santa Cruz Biotechnology, sc-8019), and total S6 (1:5000; Cell Signaling Technology, Danvers, MA, USA; #2217). HRP-conjugated goat anti-rabbit IgG (1:5000; Jackson ImmunoResearch, West Grove, PA, USA; 111-035-045) and goat anti-mouse IgG (1:5000; Jackson ImmunoResearch, West Grove, PA, USA; 115-035-062) were used as secondary antibodies.

The membrane was later incubated with anti-goat IgG, anti-mouse IgG, or anti-rabbit IgG coupled to horseradish peroxidase, and bands were detected using Immobilon Western Chemiluminescent HRP Substrate (EMD Millipore, Darmstadt, Germany). Actin was used as the loading control for initial experiments, whereas, total ribosomal protein S6 (T-S6) was used in subsequent experiments. T-S6 was selected to minimize potential bias associated with possible nonspecific or indirect effects of shRNA-mediated gene silencing on actin expression. Equal T-S6 expression across samples confirmed comparable protein loading and transfer efficiency.

### 2.8. Quantitative Real-Time RT-PCR

Total cellular RNAs were extracted using TRIzol™ Reagent (Invitrogen, Carlsbad, CA, USA) according to the manufacturer’s instructions. For cDNA synthesis, 3 μg of total RNA was reverse transcribed using random hexamer primers and reverse transcriptase. Quantitative real-time PCR (qRT-PCR) was performed using TB Green^®^ (SYBR Green) Premix Ex Taq™ (TaKaRa Bio Inc., Kusatsu, Shiga, Japan) according to the manufacturer’s protocol on a LightCycler^®^ Machine (Roche, Mannheim, Germany). PCR reactions were run in triplicate for each sample, and the expression level of 18S rRNA was used as an internal control [30]. The primer sequences used in this study are listed in Table S1.

### 2.9. Statistical Analysis

All experiments were performed in triplicate and repeated at least three times independently. Data are presented as the mean ± standard error of the mean (SEM). Statistical analyses were performed using SPSS version 11.5 (SPSS Inc., Chicago, IL, USA). Comparisons among multiple groups were analyzed using one-way analysis of variance (ANOVA) followed by Dunnett’s post hoc test, whereas comparisons between two groups were performed using a two-tailed Student’s *t*-test. A *p*-value of <0.05 was considered statistically significant.

## 3. Results

### 3.1. HIPK2 Expression Is Elevated During Adipogenesis in 3T3-L1 Cells

**Figure 1:**
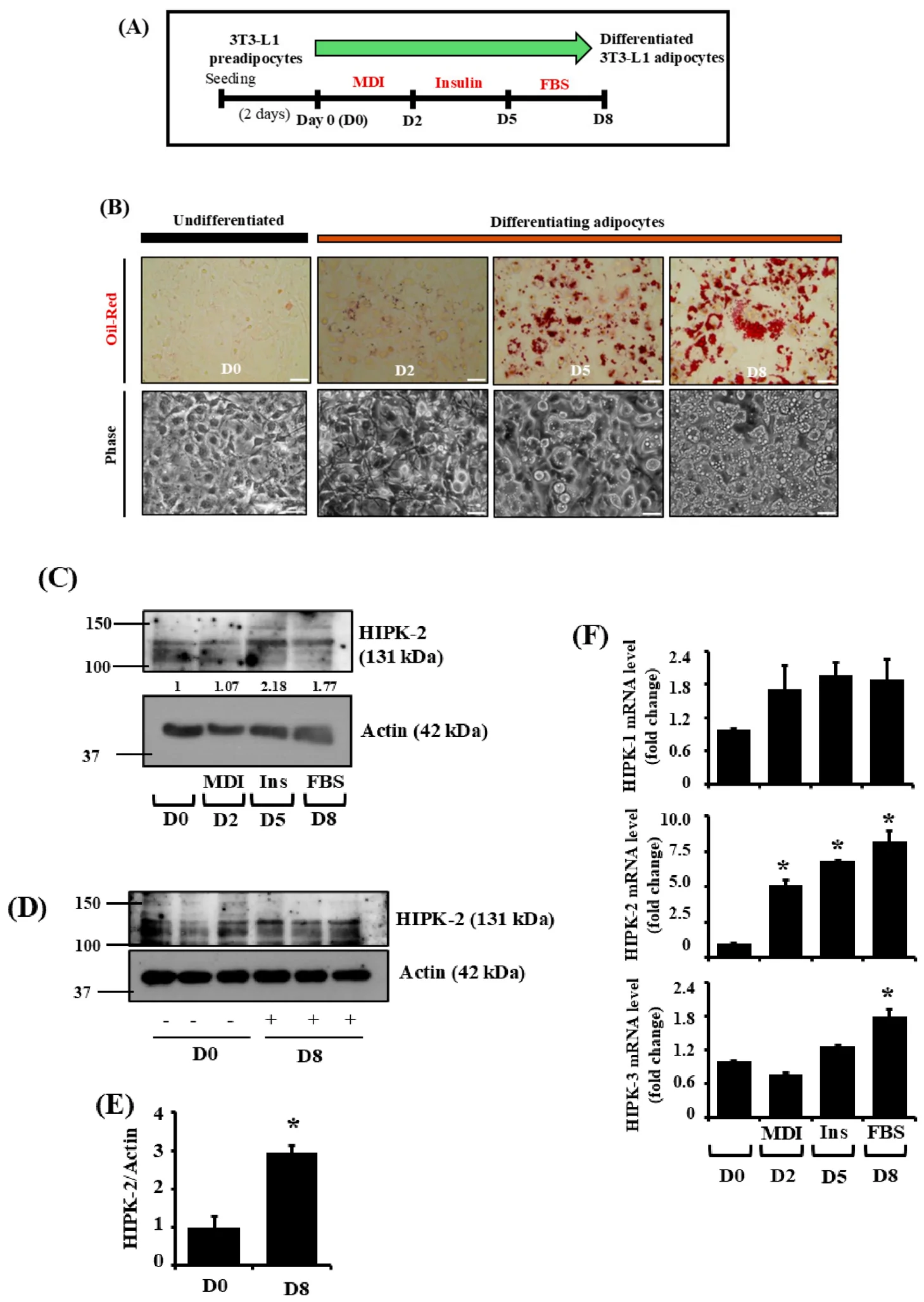
Expansion of intracellular lipid droplets (LDs) and HIPK2 expression during 3T3−L1 preadipocyte maturation. (**A**) Timeline of 3T3−1 preadipocyte differentiation. (**B**) Confirmation of lipid droplets by Oil Red O (ORO) staining and microscopic analysis at various days of 3T3−L1 differentiation. Magnification, 40× (scale bar, 20 µm). (**C**) HIPK2 protein levels at various days of 3T3-L1 differentiation. (**D**) Triplicate experiments confirming HIPK2 protein levels at day 0 and day 8. (**E**) Quantification of (**D**). (**F**) After total RNA extraction, qPCR was performed using the indicated primers. Data are presented as mean ± SE from three independent experiments. * *p* < 0.05 vs. control (D0).

Figure 1A illustrates the timeline of 3T3-L1 preadipocyte differentiation. Oil Red O (ORO) staining was commonly used to study the accumulation of intracellular lipid aggregates during 3T3-L1 preadipocyte development. On D8, 3T3-L1 cells exhibited increased intracellular lipid droplet (LD) accumulation compared to undifferentiated cells (Figure 1B, upper). Similarly, phase-contrast microscopy also revealed increased intracellular LD on D8 of adipogenesis (Figure 1B, lower). As indicated in Figure 1C, HIPK2 protein levels were low in undifferentiated 3T3-L1 preadipocytes on D0 and increased significantly during differentiation. The triplicate experiments confirmed elevated HIPK2 protein levels on D8 compared to D0 of differentiation. The densitometry data of Figure 1D are indicated in Figure 1E. As shown in Figure 1F, there was significant fold induction of HIPK2 transcripts during differentiation (D2, D5, and D8) compared with undifferentiated cells (D0), indicating a potential role of HIPK2 in adipogenesis and lipid accumulation in 3T3-L1 cells. However, mRNA levels of HIPK1 and HIPK3 remained unchanged during the differentiation process.

### 3.2. p38 MAPK, JNK 1/2, PI3K, and PKC Control HIPK2 Transcripts During 3T3-L1 Preadipocyte Differentiation

**Figure 2:**
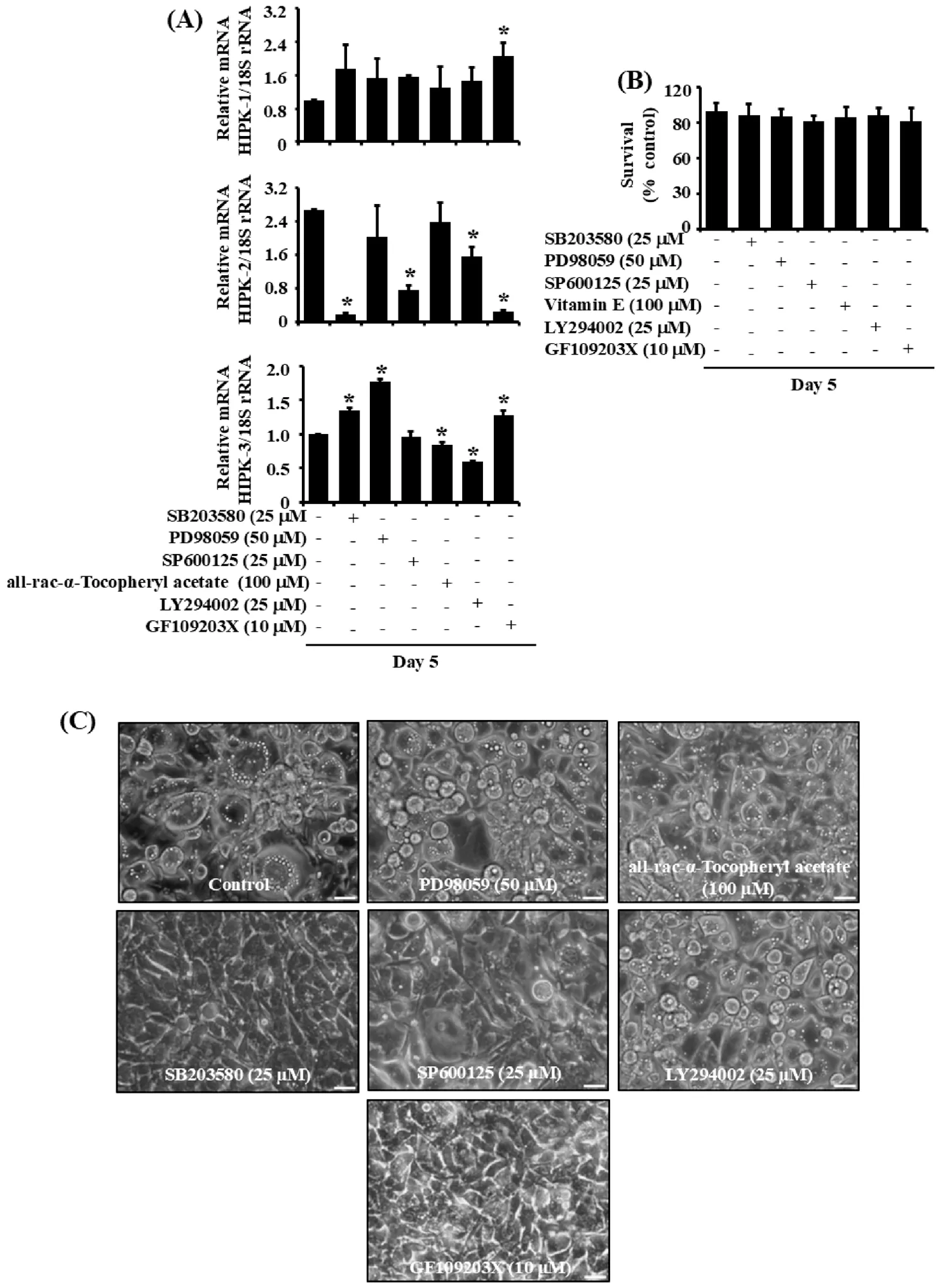
Effects of SB203580, PD98059, SP600125, all-rac-α-Tocopheryl acetate, LY294002, or GF109203X on HIPK2 transcripts in differentiating 3T3-L1 cells. (**A**) During differentiation, 3T3-L1 preadipocytes were treated with the indicated drugs until day 5 (D5). After total RNA extraction, qPCR was performed using the indicated primers. Data are presented as mean ± SE, with experiments performed independently three times; * *p* < 0.05 vs. control. (**B**,**C**) On D5, live cells were counted by trypan blue dye exclusion in control or different drug-treated groups (**B**) or morphological analysis of cells (**C**). Magnification, 40× (scale bar, 20 µm). Data are presented as mean ± SE, with experiments performed independently three times; * *p* < 0.05 vs. control (D0).

Next, we examined the signaling pathways potentially responsible for the induction of HIPK2 expression during 3T3-L1 adipogenesis. The 3T3-L1 preadipocytes were induced to differentiate with or without different inhibitors such as SB203580 (p38 MAPK), PD98059 (MEK-1/2), SP600125 (a JNK-1/2), LY294002 (a PI3K/PKB), all-rac-α-Tocopheryl acetate (an antioxidant), or GF109203X (a PKC) for 5 days. Notably, as depicted in Figure 2A, the presence of SB203580, SP600125, LY294002, and GF109203X significantly lowered the HIPK2 transcripts on D5 of differentiation. PD98059 or all-rac-α-Tocopheryl acetate did not affect HIPK2 mRNA levels. In Figure 2B, the cell count analysis reveals that treatment with different pharmacological inhibitors had no cytotoxic effects. In addition, D5 morphological analysis showed that SB203580, SP600125, LY294002, and GF109203X mitigated the LD formation in differentiated 3T3-L1 cells. These findings indicate that the elevation of HIPK2 transcripts throughout the 3T3-L1 preadipocyte differentiation is largely mediated by the p38 MAPK, JNK, PI3K, and PKC signaling pathways.

### 3.3. Depletion of HIPK2 Drives Lower Lipid Storage and Triglyceride Levels During 3T3-L1 Preadipocyte Differentiation

**Figure 3:**
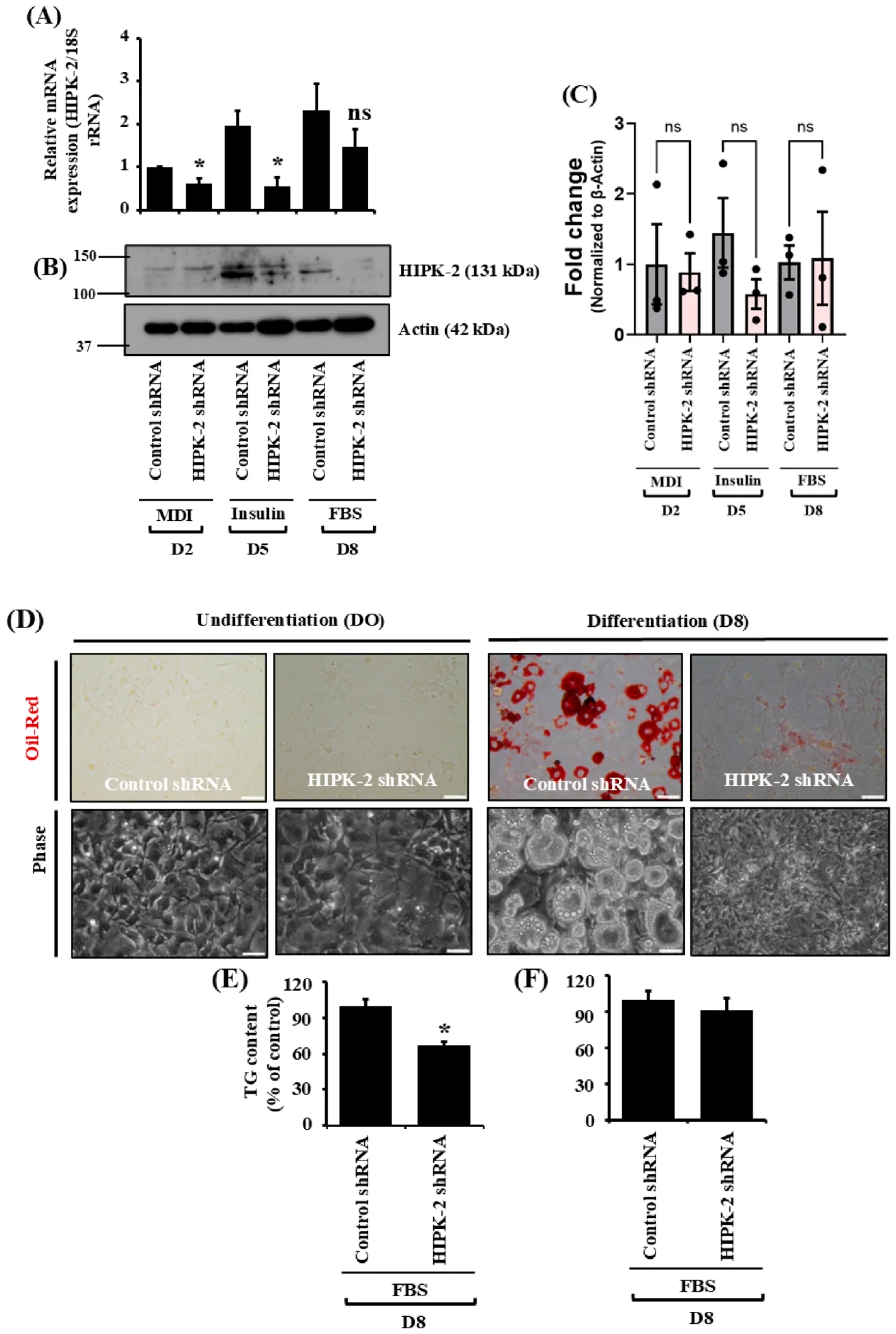
Effects of HIPK2 depletion on fat storage, triglyceride levels, and survival during adipogenesis. (**A**) Control or HIPK2-shRNA-transfected cells were differentiated, and total transcripts or proteins were harvested at different time points. Real-time qPCR. (**B**) Representative Western blot showing HIPK2 and β-actin protein expression in control-siRNA- and HIPK2-siRNA-transfected cells at Days 2, 5, and 8 of differentiation. (**C**) Densitometric quantification of HIPK2 protein levels normalized to β-actin from independent experiments. Data are presented as mean ± SE, with experiments performed independently three times; * *p* < 0.05 vs. control (D0). (**D**–**F**) On D8, differentiated control or HIPK2-shRNA-transfected cells were stained with ORO and visualized by microscopic analysis (**C**). Magnification, 40× (scale bar, 20 µm). At the same time, triglyceride levels were quantified (**D**). On D8, live cells were counted by trypan blue dye exclusion in the control and HIPK2-shRNA-transfected groups. Data are presented as mean ± SE, with experiments performed independently three times; * *p* < 0.05 vs. control, ns; non-significant.

Next, we investigated the biological function of HIPK2 in 3T3-L1 preadipocyte differentiation by using HIPK2 shRNA transfection. HIPK2-shRNA-transfected cells had lower expression of HIPK2 mRNA on D2 and D5 and protein levels on D5 or D8 in comparison to control-shRNA-transfected 3T3-L1 cells (Figure 3A,B). Importantly, HIPK2 depletion reduced lipid body deposition and triglyceride content in 3T3-L1 cells on D8 compared with control-shRNA-transfected cells (Figure 3C,D). Furthermore, D8 cell count analysis indicated that HIPK2 depletion did not affect 3T3-L1 cell survival compared with control-shRNA-transfected cells (Figure 3E). Our findings suggest that HIPK2 promotes fat accumulation during adipogenesis in 3T3-L1 cells.

### 3.4. Ablation of HIPK2 Downregulates Adipogenic Transcription Factors (C/EBP-α, PPAR-γ, and STAT-3) Throughout the Adipogenesis

**Figure 4:**
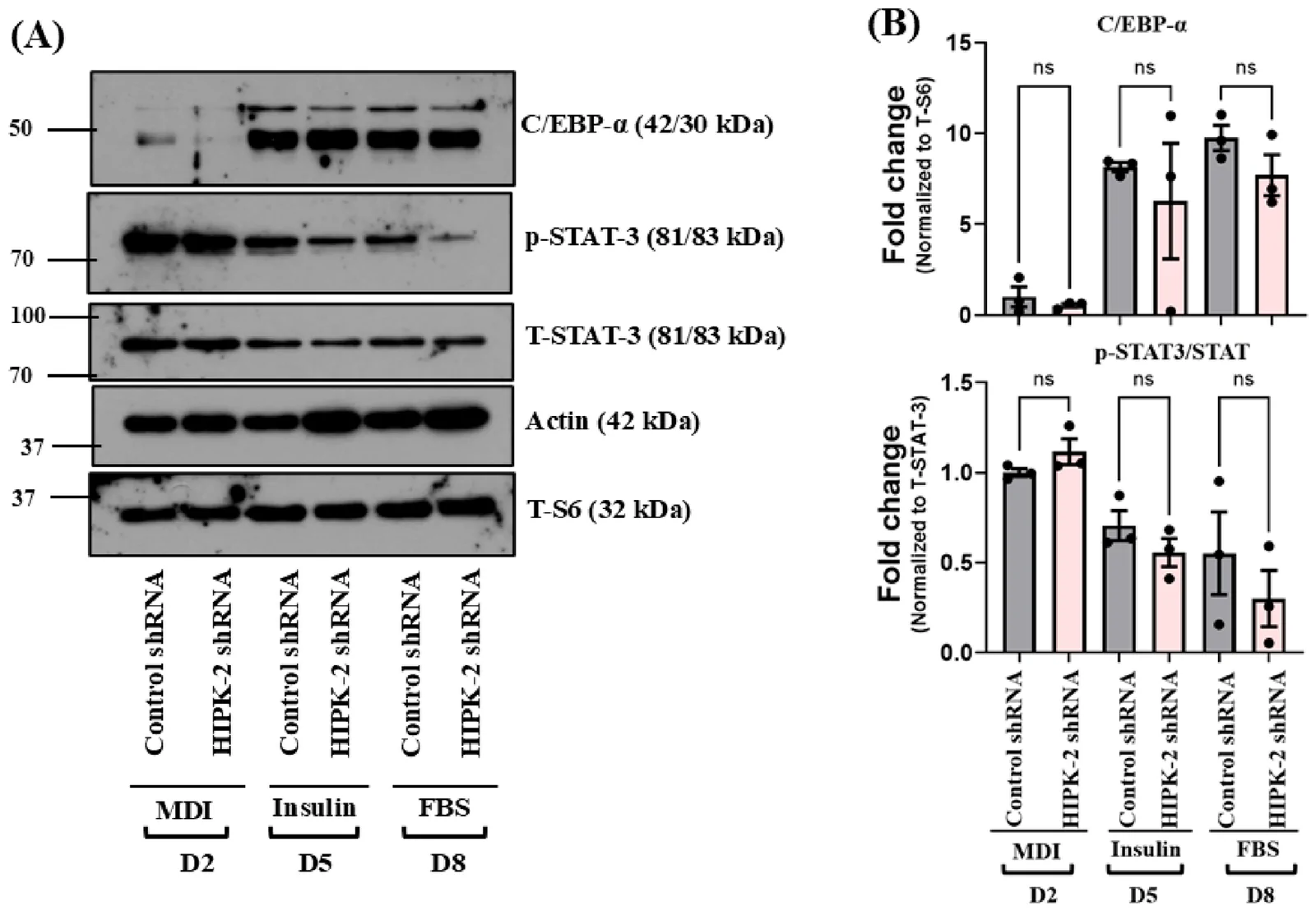

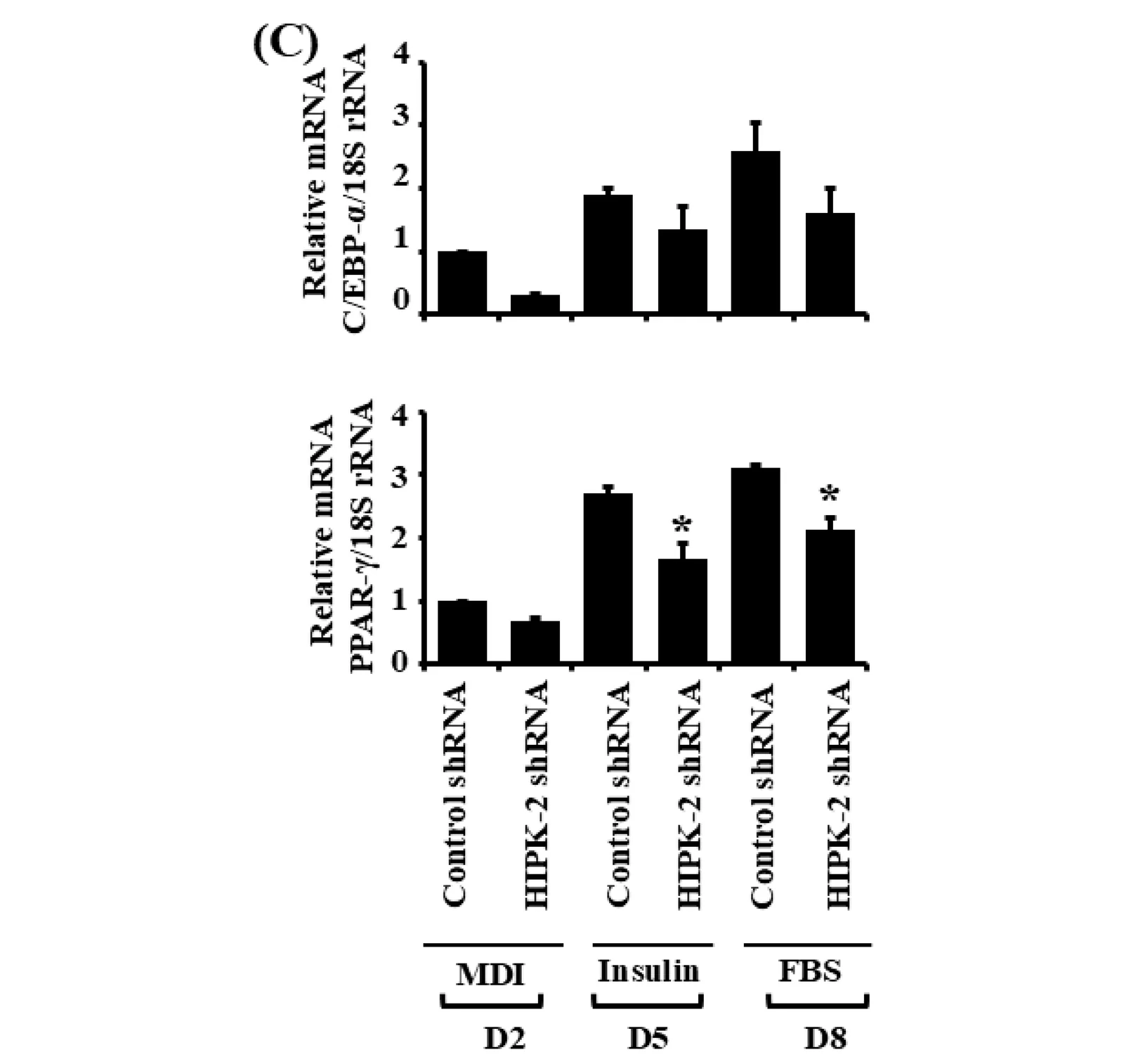
Effects of HIPK2 depletion on expression of C/EBP-α and STAT-3 during adipogenesis. (**A**,**B**) Control or HIPK2-shRNA-transfected cells were differentiated, and protein was harvested at different time points. Western blot was probed with the indicated antibodies (**A**) and densitometric quantification of (**A**) normalized to T-S6 or STAT-3 from independent experiments. (**C**) After total RNA extraction, qPCR was performed using the indicated primers. Data are presented as mean ± SE, with experiments performed independently three times; * *p* < 0.05 vs. control on the respective day, ns; non-significant.

To further investigate the mechanism by which HIPK2 regulates adipogenesis, we examined the effects of HIPK2 knockdown on the expression of the key adipogenic transcription factors C/EBPα, PPARγ, and STAT3 during the differentiation of 3T3-L1 cells [9,10,11,12,30]. Compared with control cells, HIPK2-shRNA-transfected cells showed reduced C/EBP-α protein levels on D5 and D8 (Figure 4A). The phosphorylation of STAT-3 on D5 and D8 was also reduced in HIPK2-shRNA-transfected 3T3-L1 cells compared to control-shRNA-transfected cells. S6 and total STAT-3 were not changed. Real-time qPCR showed lower transcript levels of C/EBP-α and PPAR-γ in HIPK2-shRNA-transfected 3T3-L1 cells compared with controls. These findings indicate that the reduction in C/EBP-α and PPAR-γ in adipocyte cells occurs when HIPK2 is knocked down, a process that governs adipogenesis.

### 3.5. Depletion of HIPK2 Downregulates FAS and Perilipin A Expression During 3T3-L1 Preadipocyte Differentiation

**Figure 5:**
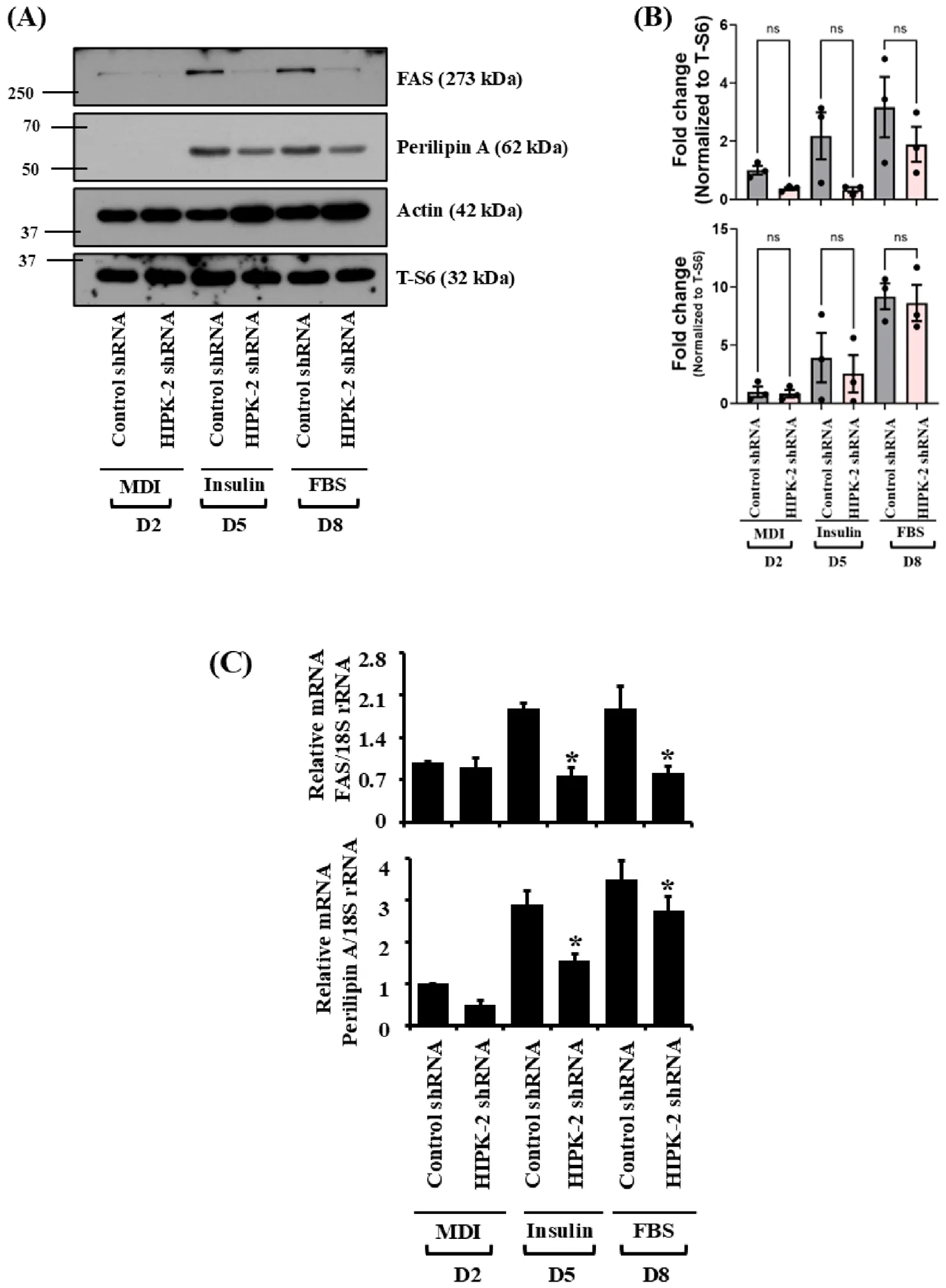
Effects of HIPK2 ablation on the protein and mRNA levels of FAS and perilipin A during adipogenesis. (**A**,**B**) Control or HIPK2-shRNA-transfected cells were differentiated, and total proteins were harvested at different time points. Western blot analysis (**A**) and densitometric quantification of (**A**) normalized to T-S6 from independent experiments. (**B**,**C**) Real-time qPCR was performed for the detection of FAS and perilipin A. Data are presented as mean ± SE, with experiments performed independently three times; * *p* < 0.05 vs. control on the respective day, ns; non-significant.

Further, throughout adipogenesis, we examined the expression of FAS [13] and perilipin A [14] in HIPK2-shRNA- or control-shRNA-transfected 3T3-L1 cells. HIPK2-shRNA-transfected cells showed a strong decrease in FAS protein levels and a slight decrease in perilipin A protein levels compared with control cells (Figure 5A). S6 was not changed. Real-time qPCR also confirmed lower transcript levels of FAS and perilipin A in HIPK2-shRNA-transfected 3T3-L1 cells compared to control cells. These findings collectively indicate that HIPK2 depletion reduces fatty acid synthase mRNA and protein levels during adipogenesis.

### 3.6. Knockdown of HIPK2 Causes the Reduced Expression of Adiponectin, Leptin, and Resistin During 3T3-L1 Preadipocyte Differentiation

**Figure 6:**
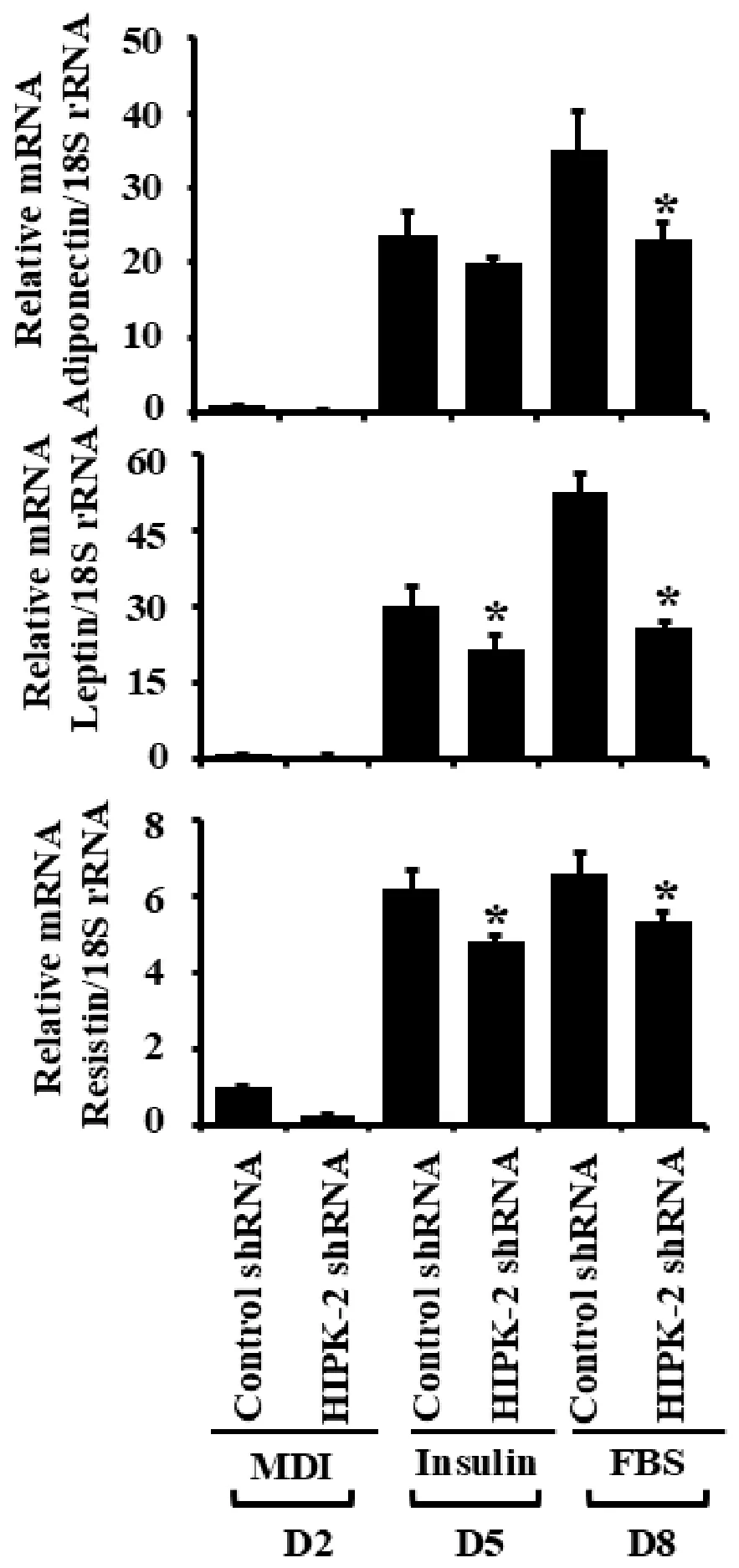
Effects of HIPK2 depletion on the transcripts of adiponectin, leptin, and resistin during 3T3-L1 preadipocyte differentiation. Control and HIPK2-shRNA-transfected cells were differentiated, and the total transcripts were extracted at different time points. Real-time qPCR was performed to detect adiponectin, leptin, and resistin. Data are presented as mean ± SE, with experiments performed independently three times; * *p* < 0.05 vs. control on the respective day.

Adipokines (adiponectin, leptin, and resistin) are involved in the pathogenesis of obesity [25,26]. Notably, real-time qPCR showed lower adiponectin transcript levels on D8 and leptin transcript levels on D5 and D8 in HIPK2-shRNA-transfected 3T3-L1 cells compared with control cells (Figure 6). Also, resistin transcript levels were significantly lower in HIPK2-shRNA-transfected 3T3-L1 cells compared with the control throughout adipogenesis.

## 4. Discussion

Excessive preadipocyte differentiation and the resultant high-fat accumulation in the adipose tissue are closely linked to the development of obesity. As mentioned above, HIPK2 has been implicated in the regulation of several transcription factors that play pivotal roles in embryonic development, apoptosis, cell proliferation, and tumor development [19,21,22,23,24,31,32]. Recent evidence has indicated that HIPK2 is an essential regulator of adipocyte differentiation and adipose tissue development in vivo and in vitro [33]. However, the regulation, expression, and function of HIPK2 during adipogenesis remain largely unknown. In this study, we demonstrated that HIPK2 expression is highly upregulated during murine 3T3-L1 white preadipocyte differentiation in p38 MAPK-, JNK-, PI3K-, and PKC-dependent manners, and that the elevated HIPK2 plays a pivotal role in fat deposition and storage during murine white 3T3-L1 preadipocyte differentiation. Depletion of HIPK2 inhibited lipid body aggregation and reduced triglyceride levels during adipogenesis in 3T3-L1 cells. We established that these effects are modulated via C/EBP-α, PPAR-γ, STAT-3, FAS, and perilipin A.

Previously, it has been reported that HIPK2 expression is high in both white adipose tissue (WAT) and brown adipose tissue (BAT) in mice [33]. There is further evidence that HIPK2 expression is low in 3T3-L1 preadipocytes but is increased in differentiated or mature adipocytes, suggesting a link between HIPK2 overexpression and white preadipocyte differentiation. Although HIPK family members share structural similarities, our expression analysis demonstrated that HIPK2 was selectively upregulated during 3T3-L1 adipocyte differentiation, whereas HIPK1 and HIPK3 expression levels remained largely unchanged during the differentiation process. In addition, previous studies have identified HIPK2 as an important regulator of adipose tissue development and adipocyte differentiation in vivo and in vitro [33]. These observations provided the rationale for focusing primarily on HIPK2 in the current study. Nevertheless, the potential compensatory or context-dependent roles of other HIPK family members during adipogenesis cannot be excluded and warrant further investigation.

However, the molecular mechanisms underlying the regulation of HIPK2 expression during adipogenesis remain enigmatic. Importantly, the outcomes of pharmacological suppression demonstrated that the HIPK2 transcript levels during adipogenesis of 3T3-L1 cells were significantly suppressed by SB203580 (a p38 MAPK inhibitor), GF109203X (a PKC inhibitor), SP600125 (a JNK-1/2 inhibitor), or LY294002 (a PI3K/PKB inhibitor). Importantly, during 3T3-L1 preadipocyte differentiation, SB203580, GF109203X, SP600125, and LY294002 were not cytotoxic. These results suggest that p38 MAPK, PKC, JNK, and PI3K signaling may contribute to the transcriptional induction of HIPK2 during 3T3-L1 preadipocyte differentiation. Further genetic studies are required to confirm their direct regulatory roles. Substantial evidence indicates that HIPK2 expression is regulated at the post-transcriptional (mRNA stability), translational, protein stability, and post-translational modification (PTM) levels [34,35]. Given that p38 MAPK, PKC, JNK-1/2, and PI3K/PKB also participate in the regulation of post-transcription (mRNA stability), translation, protein stability, and PTM of many proteins [36,37,38,39], it will be interesting to investigate in the future their role in mRNA stability, translation, protein stability, and/or PTM of HIPK2 during white preadipocyte differentiation. Although pharmacological inhibitors are widely used to study adipogenic signaling pathways, reliance solely on chemical inhibitors is a limitation of this study because of potential off-target effects. Future genetic validation studies using siRNA- or shRNA-mediated knockdown approaches will be important to further confirm the involvement of these signaling pathways in HIPK2 regulation during adipogenesis.

Recent studies have demonstrated that loss of HIPK2 specifically inhibits white adipocyte differentiation and tissue development [33]. Furthermore, gene silencing of HIPK2 blocks fat deposition during adipogenesis in 3T3-L1 cells [33]. In the current study, knockdown of HIPK2 suppressed lipid body deposition and reduced triglyceride levels during 3T3-L1 white preadipocyte differentiation. These results indicate that HIPK2 expression and activity promote adipogenesis during 3T3-L1 preadipocyte differentiation. Until now, the molecular and signaling mechanisms by which HIPK2 stimulates adipogenesis during 3T3-L1 preadipocyte differentiation remain poorly defined.

Several studies support the important function of numerous transcription factors, such as C/EBP-α, PPAR-γ, and STAT-3, in 3T3-L1 white preadipocyte differentiation. At present, the regulation of C/EBP-α, PPAR-γ, and STAT-3 by HIPK2 during 3T3-L1 white preadipocyte differentiation is unknown [9,10,11,12]. Thus, the effect of HIPK2 knockdown on the expression of C/EBP-α, PPAR-γ, and STAT-3 in the 3T3-L1 white preadipocyte differentiation process was further investigated in the current study. Of interest, depletion of HIPK2 was found to repress expression of C/EBP-α and PPAR-γ at both protein and mRNA levels in the 3T3-L1 white preadipocyte differentiation process. These results show that HIPK2 expression and activity are necessary for the transcriptional upregulation of C/EBP-α and PPAR-γ during cell differentiation. Previous studies have demonstrated that HIPK2 directly interacts with and cooperates with C/EBP-β and p300, a transcriptional coactivator, at the protein level, and that this interaction is functionally relevant and crucial for adipocyte differentiation in 3T3-L1 cells [40]. However, at this time, it is unclear how HIPK2 controls the transcription of C/EBP-α and PPAR-γ during the differentiation of 3T3-L1 white preadipocytes. Considering that HIPK2 directly interacts and cooperates with C/EBP-β, and that C/EBP-β is involved in the transcriptional up-regulation of C/EBP-α and PPAR-γ during the 3T3-L1 white preadipocyte differentiation process [40,41]. It will be interesting to test, in the future, whether gene silencing of HIPK2 leads to the reduced expression and/or transcriptional activity of C/EBP-β during the 3T3-L1 white preadipocyte differentiation process, or knock-down of C/EBP-β causes the transcriptional repression of C/EBP-α and PPAR-γ during the cell differentiation process. Previously, it has also been reported that the STAT family, including STAT-1, STAT-3, STAT-5, and STAT-6, is expressed in both 3T3-L1 preadipocytes and adipocytes and is critical for 3T3-L1 adipocyte differentiation [42]. Recently, it has been reported that HIPK2 is a positive regulator of the JAK/STAT pathway during development and tumorigenesis [43,44]. This notion is further supported by the fact that the transcriptional output of JAK/STAT signaling is perturbed upon the loss or knockdown of HIPK2. Conversely, increased HIPK2 activity elevates JAK/STAT signaling, including STAT-3, in a cell-autonomous manner [45]. In the current study, HIPK2 depletion partially inhibited STAT-3 phosphorylation without altering total STAT-3 protein levels on D5 and D8 of adipogenesis, suggesting that HIPK2 knockdown reduced the phosphorylation of pre-existing STAT-3 protein without altering de novo protein synthesis. Collectively, these data indicate that HIPK2 expression and activity also facilitate the elevated phosphorylation of STAT-3 during adipocyte differentiation. These findings suggest that reduced STAT-3 phosphorylation may be associated with the impaired adipogenic phenotype observed following HIPK2 knockdown. However, the present study does not establish whether the observed reduction in STAT-3 phosphorylation following HIPK2 depletion reflects a direct regulatory interaction or an indirect consequence of impaired adipocyte differentiation and altered adipogenic signaling. Further studies are warranted to clarify the precise relationship between HIPK2 and STAT-3 during adipogenesis.

It has been well documented that the expression of both FAS and perilipin A is largely increased during adipogenesis [13,14]. FAS is a crucial lipogenic enzyme that catalyzes intermediate steps in the biosynthesis of long-chain fatty acids, and its overexpression leads to induced fatty acid biosynthesis in cells and/or tissues [13,46]. Perilipin A binds to and stabilizes the newly formed LD during 3T3-L1 preadipocyte differentiation [14]. Substantial evidence also indicates that perilipin A is a highly phosphorylated protein in adipocytes that is not secreted but localized at the surface of LD [14]. It has also been shown that perilipin A expression is elevated in human adipose tissue [47]. Considering the roles of FAS and perilipin A in lipogenesis and LD stabilization and storage in adipocytes, blocking their activity and/or expression is likely another way to prevent or treat obesity in the cellular context of lipogenesis and LD metabolism. Little is known about HIPK2 regulation of FAS and perilipin A expression in adipocytes. Notably, in this study, knockdown of HIPK2 decreased the expression levels of FAS and perilipin A during 3T3-L1 preadipocyte differentiation. These results point out that HIPK2 expression plays a crucial role in the transcriptional and translational up-regulation of FAS and perilipin A in the 3T3-L1 preadipocyte differentiation process, and thus suppression of lipid accumulation by knockdown of HIPK2 herein is closely linked to the reduced expression of FAS and perilipin A. Adipocytes, the most abundant cell type in adipose tissue, are a major source of adipokines including adiponectin, leptin, and resistin. Reportedly, while adiponectin expression levels decrease with an increase in adiposity [48], leptin and resistin levels increase in obesity [49]. The adipocyte-derived hormone resistin is postulated to be linked to obesity, insulin resistance, and diabetes [50]. Consequently, suppressing adipokine expression, such as leptin or resistin, is an alternative to obesity. In this study, knockdown of HIPK2 inhibited the mRNA expression of adiponectin, leptin, and resistin during 3T3-L1 preadipocyte differentiation. These results suggest that HIPK2 expression and activity are crucial for the transcriptional upregulation of these adipokines during 3T3-L1 preadipocyte differentiation.

**Figure 7:**
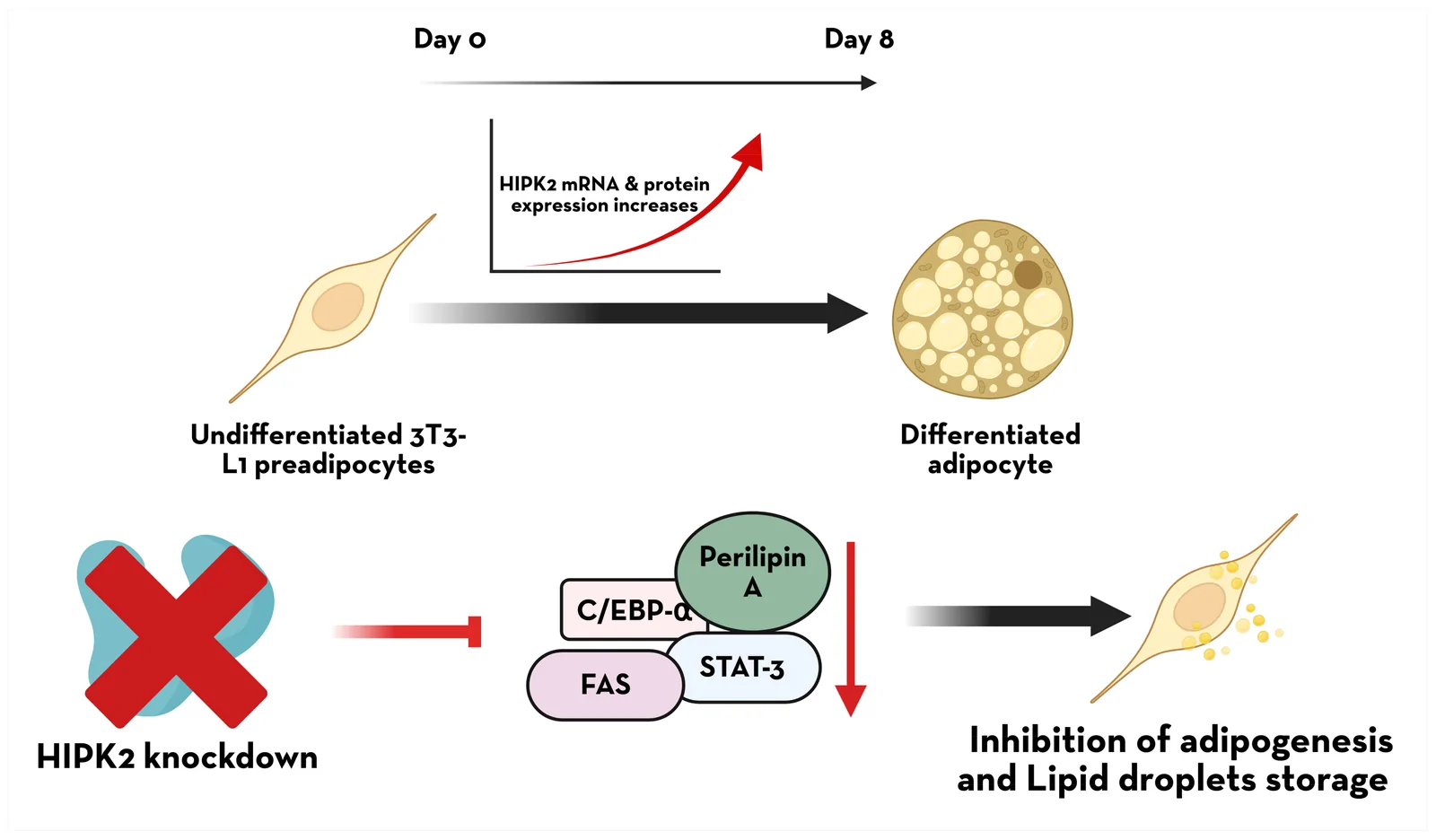
Graphical Abstract: The silencing of HIPK2 blocks adipogenesis during 3T3-L1 preadipocyte differentiation (Created in BioRender. Yadav, A.K. (2026). https://BioRender.com/q5f66e6 (accessed on 5 February 2026)).

Despite the strengths of this study, several limitations should be acknowledged. First, Western blotting is a semi-quantitative technique that is influenced by antibody specificity, protein loading, transfer efficiency, and signal detection. Although validated antibodies, appropriate loading controls, and independent biological replicates were used to improve data reliability, variability associated with antibody performance and nonspecific background cannot be completely excluded. Second, the pharmacological inhibitors used in this study may exert dose-dependent off-target effects. To minimize this limitation, inhibitor-based observations were supported by complementary HIPK2 knockdown experiments, which yielded consistent results and strengthened the proposed mechanism. Third, adiponectin, leptin, and resistin expression was evaluated at the mRNA level by RT-qPCR. Because mRNA abundance does not always correlate with protein expression or secreted protein levels, future studies should validate these findings using protein-based approaches, such as ELISA or Western blotting, together with functional analyses. Finally, the present study was performed primarily using the murine 3T3-L1 adipocyte differentiation model, which may not fully reflect the complexity of adipose tissue biology in vivo. Validation in primary adipocytes, animal models, and human adipose tissues will be necessary to confirm the physiological and clinical relevance of HIPK2 in adipogenesis and adipokine regulation. Despite these limitations, the concordant molecular, biochemical, and functional findings provide strong evidence that HIPK2 is an important regulator of adipocyte differentiation (Figure 7).

## 5. Conclusions

In summary, our findings indicate that HIPK2 expression is increased during 3T3-L1 adipocyte differentiation through p38 MAPK-, JNK-, PI3K-, and PKC-dependent signaling pathways. HIPK2 modulation was associated with changes in the expression and phosphorylation of C/EBP-α, PPAR-γ, STAT-3, FAS, and perilipin A, accompanied by altered lipid accumulation in differentiating 3T3-L1 cells. These findings suggest that HIPK2 may contribute to the regulation of adipogenesis. However, further mechanistic investigations and validation in primary cells and in vivo models are required to establish its physiological and potential therapeutic relevance.

## Data Availability

The original contributions presented in this study are included in the article/Supplementary Material. Further inquiries can be directed to the corresponding authors.

## Supplementary Materials

The following supporting information can be downloaded at https://www.mdpi.com/article/10.3390/cimb48080812/s1.

## References

[1] Blüher M. (2019). Obesity: Global epidemiology and pathogenesis. Nat. Rev. Endocrinol..

[2] Blüher M. (2025). An overview of obesity-related complications: The epidemiological evidence linking body weight and other markers of obesity to adverse health outcomes. Diabetes Obes. Metab..

[3] Sanchis-Gomar F., Lavie C.J., Mehra M.R., Henry B.M., Lippi G. (2020). Obesity and Outcomes in COVID-19: When an Epidemic and Pandemic Collide. Mayo Clin. Proc..

[4] Lippi G., Mattiuzzi C., Sanchis-Gomar F. (2025). COVID-19 and obesity: 2025 perspective on epidemiology, pathogenesis, and public health implications. J. Lab. Precis. Med..

[5] GBD 2021 US Obesity Forecasting Collaborators (2024). National-level and state-level prevalence of overweight and obesity among children, adolescents, and adults in the USA, 1990–2021, and forecasts up to 2050. Lancet.

[6] Mota de Sá P., Richard A.J., Hang H., Stephens J.M. (2017). Transcriptional Regulation of Adipogenesis. Compr. Physiol..

[7] Parra-Peralbo E., Talamillo A., Barrio R. (2021). Origin and Development of the Adipose Tissue, a Key Organ in Physiology and Disease. Front. Cell Dev. Biol..

[8] Ghaben A.L., Scherer P.E. (2019). Adipogenesis and metabolic health. Nat. Rev. Mol. Cell Biol..

[9] Farmer S.R. (2006). Transcriptional control of adipocyte formation. Cell Metab..

[10] Li X., Zeng S., Chen L., Zhang Y., Li X., Zhang B., Su D., Du Q., Zhang J., Wang H. (2024). An intronic enhancer of Cebpa regulates adipocyte differentiation and adipose tissue development via long-range loop formation. Cell Prolif..

[11] Shao X., Wang M., Wei X., Deng S., Fu N., Peng Q., Jiang Y., Ye L., Xie J., Lin Y. (2016). Peroxisome Proliferator-Activated Receptor-γ: Master Regulator of Adipogenesis and Obesity. Curr. Stem Cell Res. Ther..

[12] Burrell J.A., Boudreau A., Stephens J.M. (2020). Latest advances in STAT signaling and function in adipocytes. Clin. Sci..

[13] Olzmann J.A., Carvalho P. (2019). Dynamics and functions of lipid droplets. Nat. Rev. Mol. Cell Biol..

[14] Sztalryd C., Brasaemle D.L. (2017). The perilipin family of lipid droplet proteins: Gatekeepers of intracellular lipolysis. Biochim. Biophys. Acta Mol. Cell Biol. Lipids.

[15] Ahmadian M., Aksu A.M., Dhillon P., Zerbel Z.J., Kelemen Y., Gbayisomore O., Gómez-Banoy N., Chen S.J., Reilly S.M. (2026). Fatty acids promote uncoupled respiration via ATP/ADP carriers in white adipocytes. Nat. Metab..

[16] Jia Y., Lacombe M., Muller C., Milhas D. (2025). Adipose tissue and androgens: The ins and outs. Adipocyte.

[17] Recinella L., Orlando G., Ferrante C., Chiavaroli A., Brunetti L., Leone S. (2020). Adipokines: New Potential Therapeutic Target for Obesity and Metabolic, Rheumatic, and Cardiovascular Diseases. Front. Physiol..

[18] Longo M., Zatterale F., Naderi J., Parrillo L., Formisano P., Raciti G.A., Beguinot F., Miele C. (2019). Adipose Tissue Dysfunction as Determinant of Obesity-Associated Metabolic Complications. Int. J. Mol. Sci..

[19] Kim Y.H., Choi C.Y., Lee S.J., Conti M.A., Kim Y. (1998). Homeodomain-interacting protein kinases, a novel family of co-repressors for homeodomain transcription factors. J. Biol. Chem..

[20] Manning G., Whyte D.B., Martinez R., Hunter T., Sudarsanam S. (2002). The protein kinase complement of the human genome. Science.

[21] Conte A., Valente V., Paladino S., Pierantoni G.M. (2023). HIPK2 in cancer biology and therapy: Recent findings and future perspectives. Cell. Signal..

[22] Pena A., Jiang L., Goncharov D., Avolio T., Baust J., Sebastiani A., Scott I., Shen Y., Lin D., Saiyed A. (2025). HIPK2 Regulates Pro-proliferative Pro-survival Phenotype of Pulmonary Smooth Muscle Cells in Pulmonary Arterial Hypertension. Am. J. Respir. Crit. Care Med..

[23] He Y., Roos W.P., Wu Q., Hofmann T.G., Kaina B. (2019). The SIAH1–HIPK2–p53ser46 Damage Response Pathway is Involved in Temozolomide-Induced Glioblastoma Cell Death. Mol. Cancer Res..

[24] Feng Y., Zhou L., Sun X., Li Q. (2017). Homeodomain-interacting protein kinase 2 (HIPK2): A promising target for anti-cancer therapies. Oncotarget.

[25] Calzado M.A., Renner F., Roscic A., Schmitz M.L. (2007). HIPK2: A versatile switchboard regulating the transcription machinery and cell death. Cell Cycle.

[26] Rinaldo C., Prodosmo A., Siepi F., Soddu S. (2007). HIPK2: A multitalented partner for transcription factors in DNA damage response and development. Biochem. Cell Biol..

[27] Yadav A.K. (2021). Expression and Function of Homeodomain-Interacting Protein Kinase-2 (HIPK-2) in White and Brown Adipocytes. Ph.D. Thesis.

[28] Yadav A.K., Jang B.C. (2021). Inhibition of Lipid Accumulation and Cyclooxygenase-2 Expression in Differentiating 3T3-L1 Preadipocytes by Pazopanib, a Multikinase Inhibitor. Int. J. Mol. Sci..

[29] Yadav A.K., Jang B.C. (2022). Anti-adipogenic and Pro-lipolytic Effects on 3T3-L1 Preadipocytes by CX-4945, an Inhibitor of Casein Kinase 2. Int. J. Mol. Sci..

[30] Wang D., Zhou Y., Lei W., Zhang K., Shi J., Hu Y., Shu G., Song J. (2010). Signal transducer and activator of transcription 3 (STAT3) regulates adipocyte differentiation via peroxisome-proliferator-activated receptor γ (PPARγ). Biol. Cell.

[31] Ann E.-J., Kim M.-Y., Yoon J.-H., Ahn J.-S., Jo E.-H., Lee H.-J., Lee H.-W., Kang H.-G., Choi D.W., Chun K.-H. (2016). Tumor Suppressor HIPK2 Regulates Malignant Growth via Phosphorylation of Notch1. Cancer Res..

[32] Chen P., Duan X., Li X., Li J., Ba Q., Wang H. (2020). HIPK2 suppresses tumor growth and progression of hepatocellular carcinoma through promoting the degradation of HIF-1α. Oncogene.

[33] Sjölund J., Pelorosso F.G., Quigley D.A., DelRosario R., Balmain A. (2014). Identification of Hipk2 as an essential regulator of white fat development. Proc. Natl. Acad. Sci. USA.

[34] Saul V.V., Schmitz M.L. (2013). Posttranslational modifications regulate HIPK2, a driver of proliferative diseases. J. Mol. Med..

[35] Gatti V., Ferrara M., Virdia I., Matteoni S., Monteonofrio L., di Martino S., Diodoro M.G., Di Rocco G., Rinaldo C., Soddu S. (2020). An Alternative Splice Variant of HIPK2 with Intron Retention Contributes to Cytokinesis. Cells.

[36] Nickless A., Cheruiyot A., Flanagan K.C., Piwnica-Worms D., Stewart S.A., You Z. (2017). p38 MAPK inhibits nonsense-mediated RNA decay in response to persistent DNA damage in noncycling cells. J. Biol. Chem..

[37] Vyas S., Faucon Biguet N., Mallet J. (1990). Transcriptional and post-transcriptional regulation of tyrosine hydroxylase gene by protein kinase C. Embo J..

[38] Alarcon-Vargas D., Ronai Z. (2004). c-Jun-NH2 kinase (JNK) contributes to the regulation of c-Myc protein stability. J. Biol. Chem..

[39] Llorente A., Arora G.K., Murad R., Emerling B.M. (2025). Phosphoinositide kinases in cancer: From molecular mechanisms to therapeutic opportunities. Nat. Rev. Cancer.

[40] Zhang Q., Nottke A., Goodman R.H. (2005). Homeodomain-interacting protein kinase-2 mediates CtBP phosphorylation and degradation in UV-triggered apoptosis. Proc. Natl. Acad. Sci. USA.

[41] Cao Z., Umek R.M., McKnight S.L. (1991). Regulated expression of three C/EBP isoforms during adipose conversion of 3T3-L1 cells. Genes. Dev..

[42] Stephens J.M., Morrison R.F., Pilch P.F. (1996). The expression and regulation of STATs during 3T3-L1 adipocyte differentiation. J. Biol. Chem..

[43] Tettweiler G., Blaquiere J.A., Wray N.B., Verheyen E.M. (2020). Hipk is required for JAK/STAT activity during development and tumorigenesis. PLoS ONE.

[44] Cheung K.L., Jaganathan A., Hu Y., Xu F., Lejeune A., Sharma R., Caescu C.I., Meslamani J., Vincek A., Zhang F. (2022). HIPK2 directs cell type-specific regulation of STAT3 transcriptional activity in Th17 cell differentiation. Proc. Natl. Acad. Sci. USA.

[45] Ohtsu N., Nobuhisa I., Mochita M., Taga T. (2007). Inhibitory effects of homeodomain-interacting protein kinase 2 on the aorta-gonad-mesonephros hematopoiesis. Exp. Cell Res..

[46] Jensen-Urstad A.P., Semenkovich C.F. (2012). Fatty acid synthase and liver triglyceride metabolism: Housekeeper or messenger?. Biochim. Biophys. Acta.

[47] Kern P.A., Di Gregorio G., Lu T., Rassouli N., Ranganathan G. (2004). Perilipin expression in human adipose tissue is elevated with obesity. J. Clin. Endocrinol. Metab..

[48] Achari A.E., Jain S.K. (2017). Adiponectin, a Therapeutic Target for Obesity, Diabetes, and Endothelial Dysfunction. Int. J. Mol. Sci..

[49] Yadav A., Kataria M.A., Saini V., Yadav A. (2013). Role of leptin and adiponectin in insulin resistance. Clin. Chim. Acta.

[50] Rajala M.W., Obici S., Scherer P.E., Rossetti L. (2003). Adipose-derived resistin and gut-derived resistin-like molecule-beta selectively impair insulin action on glucose production. J. Clin. Investig..

