# Supplementary material for: Silencing of Homeodomain-Interacting Protein Kinase 2 (HIPK2) Induces Anti-Adipogenic Effects in 3T3-L1 Adipocytes
Source: Curr Issues Mol Biol. 2026 Aug 12;48(8):812. doi: 10.3390/cimb48080812 (PMC13511672; doi:10.3390/cimb48080812)
Supplement: Supplementary file 1 [file cimb-48-00812-s001.zip › cimb-4278120-supplementary.pdf]

Table S1: Sequences of primers used for quantitative real-time qPCR.

Gene	Forward	Reverse
HIPK1	TAAACGACCTCGGAGACTGC	GAGGGCTGAGATCACTGCAT
HIPK2	CACAGGCTCAAGATGGCAGA	CCATACCTTCGTACACGGGG
HIPK3	ATCGAAGCAGAACTGCACCA	CCAAAAGTGCCACGACCAAG
C/EBP-α	TTACAACAGGCCAGGTTTCC	CTCTGGGATGGATCGATTGT
PPAR-γ	GGTGAAACTCTGGGAGATTC	CAACCATTGGGTCAGCTCTC
FAS	TTGCTGGCACTACAGAATGC	AACAGCCTCAGAGCGACAAT
Perilipin A	CTTTCTCGACACACCATGGAAAC	CCACGTTATCCGTAACACCCTTCA
Leptin	CCAAAACCCTCATCAAGACC	CTCAAAGCCACCACCTCTGT
Resistin	CCGATGAGCAGTCA CCTCCA	CAGCTGCTTCGCCTCGTCCTCCT
18S rRNA	GGTGAAGGTCGGTGTGAACG	GGTAGGAACACGGAAGGCCA
